# Di-μ-azido-bis­(μ-1,4,7,10,13,16-hexa­oxacyclo­octa­deca­ne)bis­(5,10,15,20-tetra­phenyl­porphyrinato)dicadmium­disodium

**DOI:** 10.1107/S1600536812048052

**Published:** 2012-11-30

**Authors:** Hamza Toumi, Nesrine Amiri, Mohamed Salah Belkhiria, Jean-Claude Daran, Habib Nasri

**Affiliations:** a Laboratoire de Physico-chimie des Matériaux, Université de Monastir, Faculté des Sciences de Monastir, Avenue de l’environnement, 5019 Monastir, Tunisia; b Laboratoire de Chimie de Coordination CNRS UPR 8241, 205 Route de Norbone, 31077 Toulouse Cedex 04, France

## Abstract

The asymmetric unit of the title compound, [Cd_2_Na_2_(N_3_)_2_(C_44_H_28_N_4_)_2_(C_12_H_24_O_6_)_2_], consists of one half of the dimeric complex; the tetra­nuclear mol­ecule lies about an inversion centre. The Cd^II^ atom is coordinated by the four pyrrole N atoms of the 5,10,15,20-tetra­phenyl­porphyrinate ligand and one N atom of the axial azide ligand in a square-pyramidal geometry. The azide group is also linked to the Na^I^ atom, which is surrounded by one 18-crown-6 molecule and additionally bonded to a second 18-crown-6 molecule *trans* to the azide group. The porphyrin core exhibits a major doming distortion (∼40%) and the crystal structure is stabilized by weak C—H⋯π inter­actions. The mol­ecular structure features weak intra­molecular hydrogen bonds: two O—H⋯O inter­actions within the 18-crown-6 mol­ecule and one C—H(18-crown-6)⋯N(azido) contact.

## Related literature

For the synthesis of [Cd(TPP)] (TPP = 5,10,15,20-tetraphenylporphyrinato), see: Rodesiler *et al.* (1985 ▶). For related structures, see: Byrn *et al.* (1991 ▶); Mansour *et al.* (2010 ▶); Liu *et al.* (2009 ▶). For further details of geometric distortions in related compounds, see: Jentzen *et al.* (1997 ▶).
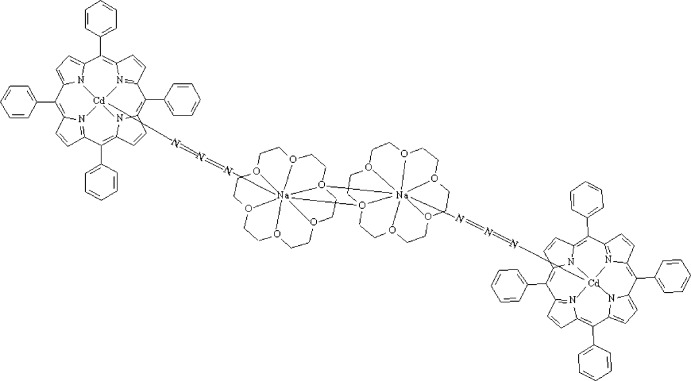


## Experimental

### 

#### Crystal data

[Cd_2_Na_2_(N_3_)_2_(C_44_H_28_N_4_)_2_(C_12_H_24_O_6_)_2_]
*M* *_r_* = 2108.90
Monoclinic, 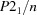

*a* = 11.4175 (3) Å
*b* = 19.5363 (4) Å
*c* = 22.6086 (6) Å
β = 102.683 (2)°
*V* = 4919.9 (2) Å^3^
*Z* = 2
Mo *K*α radiation
μ = 0.51 mm^−1^
*T* = 180 K
0.48 × 0.42 × 0.28 mm

#### Data collection

Oxford Diffraction Xcalibur (Sapphire1) diffractometer
Absorption correction: multi-scan (*CrysAlis RED*; Oxford Diffraction, 2009 ▶) *T*_min_ = 0.791, *T*_max_ = 0.870
44385 measured reflections
12338 independent reflections
9493 reflections with *I* > 2σ(*I*)
*R*_int_ = 0.030

#### Refinement

*R*[*F* ^2^ > 2σ(*F* ^2^)] = 0.027
*wR*(*F* ^2^) = 0.071
*S* = 1.02
12338 reflections
640 parameters
H-atom parameters constrained
Δρ_max_ = 0.54 e Å^−3^
Δρ_min_ = −0.39 e Å^−3^

### 

Data collection: *CrysAlis CCD* (Oxford Diffraction, 2009 ▶); cell refinement: *CrysAlis RED* (Oxford Diffraction, 2009 ▶); data reduction: *CrysAlis RED*; program(s) used to solve structure: *SIR2004* (Burla *et al.*, 2005 ▶); program(s) used to refine structure: *SHELXL97* (Sheldrick, 2008 ▶); molecular graphics: *ORTEPIII* (Burnett & Johnson, 1996 ▶) and *ORTEP-3 for Windows* (Farrugia, 2012 ▶); software used to prepare material for publication: *SHELXL97*.

## Supplementary Material

Crystal structure: contains datablock(s) I, global. DOI: 10.1107/S1600536812048052/ng5303sup1.cif

Structure factors: contains datablock(s) I. DOI: 10.1107/S1600536812048052/ng5303Isup2.hkl

Additional supplementary materials:  crystallographic information; 3D view; checkCIF report

## Figures and Tables

**Table 1:** Hydrogen-bond geometry (Å, °) *Cg*1, *Cg*2, *Cg*3 and *Cg*11 are the centroids of the N1/C11–C14, N2/C21–C24, N3/C31–C34 and C351–C356 rings, respectively.

*D*—H⋯*A*	*D*—H	H⋯*A*	*D*⋯*A*	*D*—H⋯*A*
C62—H62*A*⋯N6	0.99	2.51	3.303 (3)	137
C63—H63*B*⋯O3^i^	0.99	2.56	3.489 (2)	156
C65—H65*B*⋯O6^i^	0.99	2.47	3.257 (2)	136
C62—H62*B*⋯*Cg*1	0.99	2.90	3.555 (2)	124
C63—H63*A*⋯*Cg*11^ii^	0.99	2.75	3.662 (2)	154
C71—H71*A*⋯*Cg*3^iii^	0.99	2.88	3.535 (2)	124
C353—H353⋯*Cg*2^iv^	0.95	2.62	3.459 (2)	147
C454—H454⋯*Cg*11^iii^	0.95	2.82	3.697 (3)	153

Symmetry codes: (i) 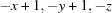; (ii) 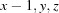; (iii) 
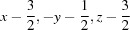
; (iv) 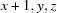.

**Fig. 1.**
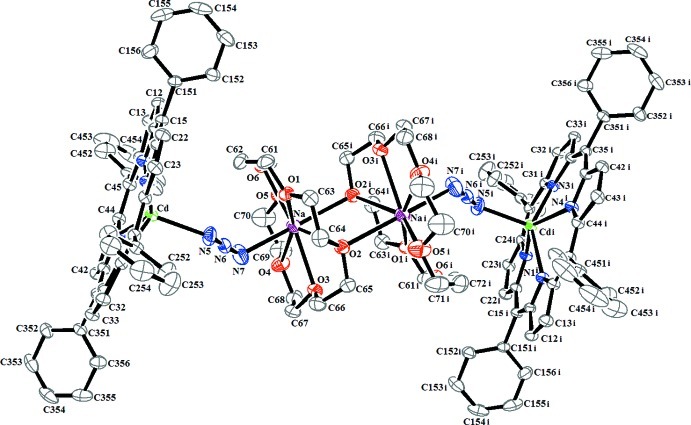
A view of the molecular structure of the title molecule with the atom-numbering. Displacement ellipsoids are drawn at 50% and H atoms have been omitted.

**Fig. 2.**
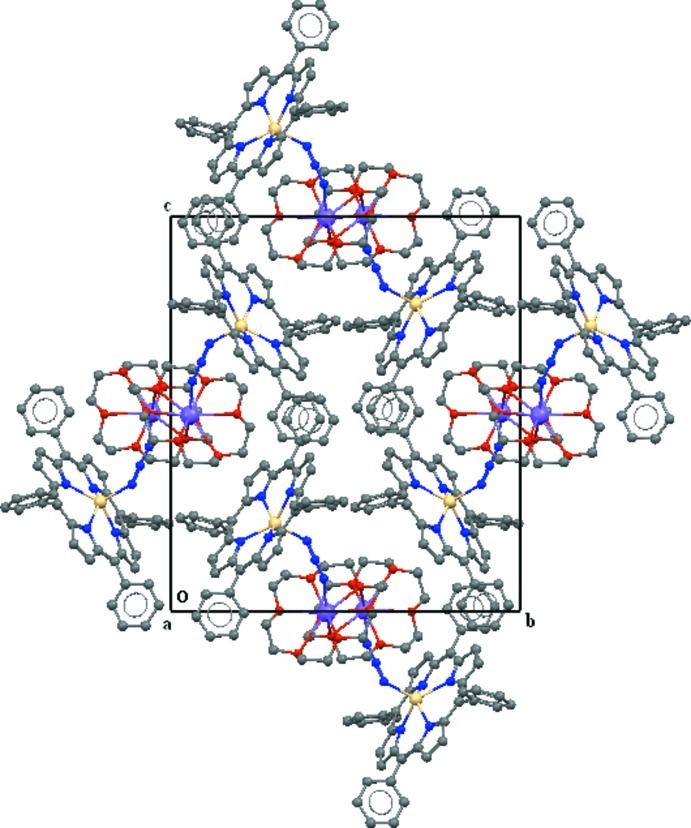
The crystal structure of the title compound plotted in projection along [100]. H atoms have been omitted.

## supplementary crystallographic information

### Comment

As part of a systematic investigation of metalloporphyrins used as biomimetic models for hemoproteines and in several other domains (*e.g.* catalysis, medicine, electronic) several metalloporphyrin complexes has been characterized by our group (Mansour *et al.*, 2010).

We report herein on the molecular structure of the title compound. The Cd atom is five-coordinated bound to the four porphyrin N atoms and to the nitrogen N5 atom of the azido ligand (Fig. 1). The Cd^__^*N*(azido) bond length [2.238 (2) Å] is in the range [2.250 (4) - 2.417 (6) Å] found for bridging µ_2_ (Cd-azido-*M*) moiety in non-porphyrin complexes where *M* is a metal atom (*i.e.*, Liu *et al.* 2009).

The most interesting features of the structure of (I) are: (i) each [Cd(TPP)(N_3_)]^-^ complex ion is strongly linked to the sodium atom of the counterion [Na(18-crown-6)]^+^ through the nitrogen N7 of the azido ligand where the Na^__^*N*(azido) distance is 2.492 (2) Å, (ii) the sodium atoms of two symmetry related [Na(18—C-6)]^+^ counterions are linked by two strong Na—O distances where the Na^+^ belongs to one 18-crown-6 molecule and the oxygen atom belong to the symmetry related adjacent ether crown molecule. The value of this distance [2.540 (1) Å] is shorter than the average Na—O(18-crown-6) which is 2.703 (2) Å.

The average equatorial cadmium-pyrrole N bond length (Cd—N_p_) [2.2148 (13) Å] is in the range [2.126 (9), 2.3167 (3) Å] for Cd porphyrin complexes (*i.e.*, Byrn *et al.*, 1991). The porphyrin core is not very distorted but present a major doming deformation as confirmed by the Normal Structural Decomposition (NSD) calculations (Jentzen, *et al.*,1997) with a value of 42%.

The molecular structure of (I) shows the presence of weak intramoleculair hydrogen bonds: two O—H···O bonds within the 18-crown-6 molecule and one *C*—*H*(18-crown-6)···N6(azido) bond. The crystal packing of the title compound is stabilized by C—H······π intermolecular interactions involving *Cg* pyrrole and phenyl centroids rings (Table 1 and Fig. 2).

### Experimental

The [Cd(TPP)] complex (Rodesiler *et al.* 1985) (20 mg, 0.027 mmol) with an excess of sodium azide NaN_3_ (100 mg, 1.5 mmol) and 18-crown-6 (52 mg, 0.19 mmol) in chlorobenzene (10 ml) were stirred overnight at room temperature under air to yield a dark-purple solution. Crystals of the title complex were obtained by diffusion of hexanes through the chlorobenzene solution.

### Refinement

All H atoms attached to C atoms were fixed geometrically and treated as riding with C—H = 0.99 Å (methylene) and 0.95 Å (aromatic) with *U*_iso_(H) = 1.2*U*_eq_(C_aromatic, methylene_).

### Crystal data

**Table:**

[Cd_2_Na_2_(N_3_)_2_(C_44_H_28_N_4_)_2_(C_12_H_24_O_6_)_2_]	*F*(000) = 2176.0
*M**_r_* = 2108.90	*D*_x_ = 1.424 Mg m^−^^3^
Monoclinic, *P*2_1_/*n*	Mo *K*α radiation, λ = 0.71073 Å
Hall symbol: -P 2yn	Cell parameters from 23347 reflections
*a* = 11.4175 (3) Å	θ = 3.0–32.2°
*b* = 19.5363 (4) Å	µ = 0.51 mm^−^^1^
*c* = 22.6086 (6) Å	*T* = 180 K
β = 102.683 (2)°	Prism, dark purple
*V* = 4919.9 (2) Å^3^	0.48 × 0.42 × 0.28 mm
*Z* = 2	

### Data collection

**Table:**

Oxford Diffraction Xcalibur (Sapphire1) diffractometer	12338 independent reflections
Radiation source: fine-focus sealed tube	9493 reflections with *I* > 2σ(*I*)
Graphite monochromator	*R*_int_ = 0.030
Detector resolution: 8.2632 pixels mm^-1^	θ_max_ = 28.4°, θ_min_ = 3.1°
ω scans	*h* = −15→15
Absorption correction: multi-scan (*CrysAlis RED*; Oxford Diffraction, 2009)	*k* = −26→26
*T*_min_ = 0.791, *T*_max_ = 0.870	*l* = −30→26
44385 measured reflections	

### Refinement

**Table:**

Refinement on *F*^2^	Primary atom site location: structure-invariant direct methods
Least-squares matrix: full	Secondary atom site location: difference Fourier map
*R*[*F*^2^ > 2σ(*F*^2^)] = 0.027	Hydrogen site location: inferred from neighbouring sites
*wR*(*F*^2^) = 0.071	H-atom parameters constrained
*S* = 1.02	*w* = 1/[σ^2^(*F*_o_^2^) + (0.041*P*)^2^] where *P* = (*F*_o_^2^ + 2*F*_c_^2^)/3
12338 reflections	(Δ/σ)_max_ = 0.002
640 parameters	Δρ_max_ = 0.54 e Å^−^^3^
0 restraints	Δρ_min_ = −0.39 e Å^−^^3^

### Special details

**Table:**

Geometry. All s.u.'s (except the s.u. in the dihedral angle between two l.s. planes) are estimated using the full covariance matrix. The cell s.u.'s are taken into account individually in the estimation of s.u.'s in distances, angles and torsion angles; correlations between s.u.'s in cell parameters are only used when they are defined by crystal symmetry. An approximate (isotropic) treatment of cell s.u.'s is used for estimating s.u.'s involving l.s. planes.
Refinement. Refinement of *F*^2^ against ALL reflections. The weighted *R*-factor *wR* and goodness of fit *S* are based on *F*^2^, conventional *R*-factors *R* are based on *F*, with *F* set to zero for negative *F*^2^. The threshold expression of *F*^2^ > σ(*F*^2^) is used only for calculating *R*-factors(gt) *etc*. and is not relevant to the choice of reflections for refinement. *R*-factors based on *F*^2^ are statistically about twice as large as those based on *F*, and *R*- factors based on ALL data will be even larger.

### Fractional atomic coordinates and isotropic or equivalent isotropic displacement parameters (Å^2^)

**Table:**

	*x*	*y*	*z*	*U*_iso_*/*U*_eq_	
Cd	0.955191 (10)	0.298901 (6)	0.222285 (5)	0.01846 (4)	
C11	0.79257 (14)	0.16615 (8)	0.18211 (7)	0.0200 (3)	
C12	0.75768 (15)	0.12022 (8)	0.13139 (7)	0.0228 (3)	
H12	0.6898	0.0908	0.1236	0.027*	
C13	0.84030 (15)	0.12703 (9)	0.09686 (8)	0.0250 (4)	
H13	0.8409	0.1034	0.0602	0.030*	
C14	0.92715 (14)	0.17697 (8)	0.12627 (7)	0.0210 (3)	
C15	0.73057 (14)	0.17493 (8)	0.22913 (7)	0.0196 (3)	
C21	0.76402 (14)	0.21780 (8)	0.27977 (7)	0.0198 (3)	
C22	0.70306 (15)	0.22250 (9)	0.32908 (7)	0.0235 (3)	
H22	0.6331	0.1981	0.3327	0.028*	
C23	0.76396 (14)	0.26804 (9)	0.36900 (7)	0.0233 (3)	
H23	0.7448	0.2816	0.4061	0.028*	
C24	0.86392 (14)	0.29252 (8)	0.34505 (7)	0.0192 (3)	
C25	0.95047 (14)	0.33965 (8)	0.37323 (7)	0.0193 (3)	
C31	1.05490 (14)	0.36012 (8)	0.35404 (7)	0.0187 (3)	
C32	1.15390 (14)	0.39757 (8)	0.39028 (7)	0.0221 (3)	
H32	1.1575	0.4163	0.4294	0.026*	
C33	1.24091 (14)	0.40109 (8)	0.35806 (7)	0.0213 (3)	
H33	1.3172	0.4223	0.3706	0.026*	
C34	1.19591 (13)	0.36672 (8)	0.30123 (7)	0.0185 (3)	
C35	1.25978 (14)	0.35663 (8)	0.25478 (7)	0.0191 (3)	
C41	1.22266 (14)	0.31638 (8)	0.20222 (7)	0.0199 (3)	
C42	1.28971 (14)	0.30580 (8)	0.15556 (7)	0.0221 (3)	
H42	1.3652	0.3255	0.1543	0.027*	
C43	1.22553 (15)	0.26289 (9)	0.11422 (7)	0.0239 (3)	
H43	1.2472	0.2469	0.0784	0.029*	
C44	1.11770 (14)	0.24567 (8)	0.13440 (7)	0.0210 (3)	
C45	1.02922 (14)	0.19925 (8)	0.10574 (7)	0.0220 (3)	
C61	0.57959 (19)	0.32908 (10)	0.09250 (9)	0.0379 (5)	
H61A	0.4970	0.3132	0.0748	0.046*	
H61B	0.5976	0.3189	0.1365	0.046*	
C62	0.6662 (2)	0.29300 (10)	0.06335 (10)	0.0400 (5)	
H62A	0.7492	0.3068	0.0826	0.048*	
H62B	0.6591	0.2429	0.0681	0.048*	
C63	0.51419 (17)	0.43920 (10)	0.11408 (8)	0.0329 (4)	
H63A	0.5162	0.4191	0.1545	0.039*	
H63B	0.4301	0.4388	0.0906	0.039*	
C64	0.56024 (19)	0.51090 (10)	0.12088 (8)	0.0372 (5)	
H64A	0.5172	0.5373	0.1469	0.045*	
H64B	0.6468	0.5109	0.1403	0.045*	
C65	0.60174 (17)	0.60662 (10)	0.06432 (10)	0.0363 (4)	
H65A	0.5969	0.6306	0.1023	0.044*	
H65B	0.5594	0.6350	0.0299	0.044*	
C66	0.73074 (18)	0.60012 (11)	0.06112 (9)	0.0392 (5)	
H66A	0.7690	0.6459	0.0649	0.047*	
H66B	0.7738	0.5712	0.0949	0.047*	
C67	0.85595 (17)	0.57584 (10)	−0.00566 (11)	0.0420 (5)	
H67A	0.9129	0.5490	0.0250	0.050*	
H67B	0.8820	0.6243	−0.0025	0.050*	
C68	0.85524 (19)	0.54956 (10)	−0.06700 (11)	0.0439 (5)	
H68A	0.7950	0.5746	−0.0975	0.053*	
H68B	0.9351	0.5562	−0.0765	0.053*	
C69	0.8252 (2)	0.44956 (13)	−0.12541 (11)	0.0554 (6)	
H69A	0.9025	0.4584	−0.1370	0.066*	
H69B	0.7600	0.4700	−0.1566	0.066*	
C70	0.8059 (3)	0.37510 (13)	−0.12149 (12)	0.0630 (7)	
H70A	0.8078	0.3530	−0.1607	0.076*	
H70B	0.8702	0.3548	−0.0898	0.076*	
C71	0.6697 (2)	0.29346 (12)	−0.09761 (12)	0.0543 (7)	
H71A	0.7117	0.2649	−0.1226	0.065*	
H71B	0.5825	0.2846	−0.1110	0.065*	
C72	0.7101 (2)	0.27356 (12)	−0.03293 (11)	0.0491 (6)	
H72A	0.6993	0.2237	−0.0282	0.059*	
H72B	0.7962	0.2846	−0.0182	0.059*	
C151	0.61833 (14)	0.13391 (8)	0.22497 (7)	0.0205 (3)	
C152	0.50758 (15)	0.16382 (9)	0.20413 (8)	0.0283 (4)	
H152	0.5028	0.2107	0.1928	0.034*	
C153	0.40297 (18)	0.12598 (12)	0.19951 (10)	0.0415 (5)	
H153	0.3270	0.1472	0.1858	0.050*	
C154	0.4095 (2)	0.05846 (12)	0.21466 (10)	0.0476 (6)	
H154	0.3379	0.0323	0.2101	0.057*	
C156	0.62280 (18)	0.06554 (9)	0.24185 (9)	0.0346 (4)	
H156	0.6982	0.0444	0.2573	0.042*	
C155	0.5182 (2)	0.02790 (10)	0.23634 (11)	0.0475 (6)	
H155	0.5219	−0.0190	0.2476	0.057*	
C251	0.93115 (14)	0.37047 (9)	0.43087 (7)	0.0217 (3)	
C252	0.89204 (19)	0.43716 (10)	0.43156 (9)	0.0361 (4)	
H252	0.8792	0.4637	0.3955	0.043*	
C253	0.8714 (2)	0.46554 (12)	0.48412 (10)	0.0491 (6)	
H253	0.8448	0.5116	0.4840	0.059*	
C254	0.8890 (2)	0.42795 (11)	0.53662 (9)	0.0426 (5)	
H254	0.8740	0.4477	0.5726	0.051*	
C255	0.92830 (18)	0.36194 (11)	0.53656 (8)	0.0357 (4)	
H255	0.9417	0.3358	0.5729	0.043*	
C256	0.94880 (17)	0.33272 (10)	0.48362 (8)	0.0315 (4)	
H256	0.9751	0.2866	0.4838	0.038*	
C351	1.37943 (14)	0.39124 (9)	0.26353 (7)	0.0213 (3)	
C352	1.48392 (15)	0.35385 (10)	0.26812 (8)	0.0266 (4)	
H352	1.4800	0.3053	0.2655	0.032*	
C353	1.59456 (16)	0.38634 (11)	0.27650 (8)	0.0368 (5)	
H353	1.6654	0.3601	0.2785	0.044*	
C354	1.60158 (18)	0.45626 (12)	0.28195 (9)	0.0429 (5)	
H354	1.6776	0.4783	0.2887	0.051*	
C355	1.4996 (2)	0.49444 (11)	0.27768 (9)	0.0397 (5)	
H355	1.5045	0.5428	0.2814	0.048*	
C356	1.38891 (16)	0.46187 (9)	0.26790 (8)	0.0302 (4)	
H356	1.3181	0.4886	0.2641	0.036*	
C451	1.04429 (15)	0.16895 (10)	0.04724 (8)	0.0280 (4)	
C452	1.0733 (2)	0.10085 (11)	0.04350 (10)	0.0447 (5)	
H452	1.0872	0.0728	0.0787	0.054*	
C453	1.0821 (2)	0.07327 (14)	−0.01290 (14)	0.0646 (8)	
H453	1.1020	0.0264	−0.0158	0.078*	
C454	1.0622 (2)	0.11344 (18)	−0.06310 (12)	0.0666 (9)	
H454	1.0686	0.0945	−0.1010	0.080*	
C455	1.0333 (2)	0.18053 (17)	−0.05956 (10)	0.0575 (7)	
H455	1.0188	0.2081	−0.0950	0.069*	
C456	1.02489 (17)	0.20883 (12)	−0.00495 (9)	0.0390 (5)	
H456	1.0057	0.2560	−0.0029	0.047*	
N1	0.89440 (12)	0.19971 (7)	0.17725 (6)	0.0217 (3)	
N2	0.86096 (12)	0.26054 (7)	0.29118 (6)	0.0203 (3)	
N3	1.08356 (11)	0.34256 (7)	0.30064 (6)	0.0187 (3)	
N4	1.11881 (12)	0.27945 (7)	0.18739 (6)	0.0217 (3)	
N5	0.85120 (16)	0.38938 (9)	0.17817 (8)	0.0395 (4)	
N6	0.85280 (14)	0.41290 (8)	0.13048 (8)	0.0376 (4)	
N7	0.85002 (16)	0.43766 (11)	0.08343 (10)	0.0583 (6)	
Na	0.67309 (6)	0.44498 (4)	−0.00329 (3)	0.03434 (17)	
O1	0.58802 (11)	0.40026 (6)	0.08330 (6)	0.0298 (3)	
O2	0.54207 (11)	0.54180 (6)	0.06255 (6)	0.0310 (3)	
O6	0.63991 (12)	0.31060 (6)	0.00076 (6)	0.0360 (3)	
O3	0.73794 (11)	0.57028 (6)	0.00506 (6)	0.0334 (3)	
O4	0.82635 (13)	0.47896 (7)	−0.06839 (6)	0.0410 (3)	
O5	0.69298 (14)	0.36427 (8)	−0.10704 (7)	0.0518 (4)	

### Atomic displacement parameters (Å^2^)

**Table:**

	*U*^11^	*U*^22^	*U*^33^	*U*^12^	*U*^13^	*U*^23^
Cd	0.01716 (6)	0.02225 (6)	0.01685 (6)	−0.00263 (5)	0.00563 (4)	−0.00278 (5)
C11	0.0196 (8)	0.0186 (8)	0.0216 (8)	−0.0015 (6)	0.0044 (6)	−0.0016 (6)
C12	0.0237 (8)	0.0204 (8)	0.0245 (8)	−0.0039 (6)	0.0053 (7)	−0.0040 (7)
C13	0.0267 (9)	0.0252 (9)	0.0235 (9)	−0.0032 (7)	0.0068 (7)	−0.0083 (7)
C14	0.0210 (8)	0.0226 (8)	0.0205 (8)	−0.0024 (6)	0.0068 (7)	−0.0049 (6)
C15	0.0188 (8)	0.0193 (7)	0.0214 (8)	−0.0026 (6)	0.0061 (6)	0.0000 (6)
C21	0.0182 (8)	0.0215 (8)	0.0208 (8)	−0.0029 (6)	0.0065 (6)	0.0004 (6)
C22	0.0207 (8)	0.0281 (9)	0.0236 (8)	−0.0038 (6)	0.0093 (7)	−0.0003 (7)
C23	0.0206 (8)	0.0309 (9)	0.0202 (8)	−0.0019 (7)	0.0086 (7)	−0.0013 (7)
C24	0.0175 (7)	0.0240 (8)	0.0171 (7)	0.0012 (6)	0.0063 (6)	−0.0020 (6)
C25	0.0199 (8)	0.0209 (8)	0.0181 (8)	0.0018 (6)	0.0060 (6)	−0.0022 (6)
C31	0.0189 (8)	0.0199 (8)	0.0177 (7)	0.0008 (6)	0.0049 (6)	−0.0025 (6)
C32	0.0245 (8)	0.0230 (8)	0.0190 (8)	−0.0022 (7)	0.0056 (6)	−0.0051 (7)
C33	0.0186 (8)	0.0235 (8)	0.0216 (8)	−0.0040 (6)	0.0036 (6)	−0.0040 (7)
C34	0.0169 (7)	0.0192 (8)	0.0196 (8)	−0.0004 (6)	0.0043 (6)	0.0009 (6)
C35	0.0162 (7)	0.0208 (8)	0.0210 (8)	−0.0006 (6)	0.0057 (6)	0.0013 (6)
C41	0.0165 (8)	0.0235 (8)	0.0207 (8)	−0.0013 (6)	0.0065 (6)	0.0002 (6)
C42	0.0202 (8)	0.0261 (9)	0.0222 (8)	−0.0016 (6)	0.0093 (6)	0.0005 (7)
C43	0.0230 (8)	0.0303 (9)	0.0209 (8)	0.0003 (7)	0.0103 (7)	−0.0016 (7)
C44	0.0208 (8)	0.0244 (8)	0.0197 (8)	0.0007 (6)	0.0085 (6)	−0.0034 (6)
C45	0.0216 (8)	0.0264 (8)	0.0196 (8)	−0.0008 (7)	0.0081 (6)	−0.0046 (7)
C61	0.0470 (12)	0.0309 (10)	0.0344 (11)	−0.0095 (9)	0.0059 (9)	0.0088 (9)
C62	0.0441 (12)	0.0273 (10)	0.0433 (12)	0.0012 (8)	−0.0017 (9)	0.0067 (9)
C63	0.0348 (10)	0.0419 (11)	0.0237 (9)	−0.0026 (8)	0.0100 (8)	0.0030 (8)
C64	0.0427 (12)	0.0448 (12)	0.0235 (9)	0.0007 (9)	0.0055 (8)	−0.0022 (8)
C65	0.0359 (11)	0.0258 (10)	0.0456 (12)	−0.0008 (8)	0.0058 (9)	−0.0055 (9)
C66	0.0362 (11)	0.0347 (11)	0.0441 (12)	−0.0065 (8)	0.0030 (9)	−0.0082 (9)
C67	0.0265 (10)	0.0329 (11)	0.0671 (15)	−0.0077 (8)	0.0113 (10)	0.0014 (10)
C68	0.0361 (11)	0.0372 (11)	0.0645 (15)	−0.0014 (9)	0.0241 (11)	0.0149 (11)
C69	0.0664 (17)	0.0668 (17)	0.0399 (13)	−0.0115 (13)	0.0267 (12)	0.0002 (12)
C70	0.0738 (18)	0.0661 (17)	0.0622 (17)	−0.0110 (14)	0.0436 (15)	−0.0202 (13)
C71	0.0617 (16)	0.0451 (14)	0.0637 (16)	−0.0151 (11)	0.0307 (13)	−0.0267 (12)
C72	0.0434 (13)	0.0357 (11)	0.0723 (17)	0.0059 (9)	0.0213 (12)	−0.0117 (11)
C151	0.0223 (8)	0.0207 (8)	0.0202 (8)	−0.0047 (6)	0.0081 (6)	−0.0027 (6)
C152	0.0246 (9)	0.0295 (10)	0.0299 (9)	−0.0050 (7)	0.0043 (7)	0.0027 (7)
C153	0.0232 (10)	0.0557 (14)	0.0441 (12)	−0.0090 (9)	0.0042 (9)	0.0019 (10)
C154	0.0401 (12)	0.0531 (14)	0.0526 (14)	−0.0289 (11)	0.0170 (10)	−0.0047 (11)
C156	0.0374 (11)	0.0237 (9)	0.0446 (12)	0.0001 (8)	0.0131 (9)	0.0022 (8)
C155	0.0630 (16)	0.0250 (10)	0.0608 (15)	−0.0171 (10)	0.0273 (12)	−0.0012 (10)
C251	0.0180 (8)	0.0285 (9)	0.0198 (8)	−0.0035 (6)	0.0068 (6)	−0.0064 (7)
C252	0.0494 (12)	0.0324 (10)	0.0289 (10)	0.0084 (9)	0.0140 (9)	−0.0026 (8)
C253	0.0714 (16)	0.0392 (12)	0.0405 (12)	0.0149 (11)	0.0205 (11)	−0.0108 (10)
C254	0.0523 (13)	0.0511 (13)	0.0305 (11)	−0.0047 (10)	0.0220 (10)	−0.0164 (10)
C255	0.0409 (11)	0.0474 (12)	0.0213 (9)	−0.0065 (9)	0.0120 (8)	−0.0019 (8)
C256	0.0366 (10)	0.0334 (10)	0.0259 (9)	−0.0001 (8)	0.0101 (8)	−0.0015 (8)
C351	0.0186 (8)	0.0284 (9)	0.0176 (8)	−0.0038 (6)	0.0055 (6)	−0.0003 (7)
C352	0.0209 (9)	0.0368 (10)	0.0220 (8)	−0.0010 (7)	0.0042 (7)	0.0051 (7)
C353	0.0185 (9)	0.0617 (14)	0.0291 (10)	−0.0010 (9)	0.0030 (7)	0.0131 (9)
C354	0.0273 (11)	0.0715 (16)	0.0281 (10)	−0.0240 (10)	0.0023 (8)	0.0087 (10)
C355	0.0465 (12)	0.0388 (11)	0.0359 (11)	−0.0222 (10)	0.0132 (9)	−0.0037 (9)
C356	0.0300 (10)	0.0295 (10)	0.0332 (10)	−0.0056 (7)	0.0115 (8)	−0.0027 (8)
C451	0.0220 (9)	0.0395 (10)	0.0254 (9)	−0.0098 (7)	0.0115 (7)	−0.0130 (8)
C452	0.0498 (13)	0.0425 (12)	0.0495 (13)	−0.0070 (10)	0.0272 (11)	−0.0187 (10)
C453	0.0583 (16)	0.0649 (17)	0.082 (2)	−0.0199 (13)	0.0394 (15)	−0.0508 (16)
C454	0.0459 (14)	0.121 (3)	0.0415 (14)	−0.0345 (15)	0.0288 (11)	−0.0446 (16)
C455	0.0363 (13)	0.113 (2)	0.0262 (11)	−0.0203 (13)	0.0136 (9)	−0.0123 (13)
C456	0.0271 (10)	0.0663 (15)	0.0259 (10)	−0.0075 (9)	0.0105 (8)	−0.0044 (9)
N1	0.0209 (7)	0.0252 (7)	0.0210 (7)	−0.0036 (6)	0.0088 (5)	−0.0059 (6)
N2	0.0191 (7)	0.0246 (7)	0.0188 (7)	−0.0038 (5)	0.0074 (5)	−0.0039 (6)
N3	0.0168 (6)	0.0221 (7)	0.0179 (7)	−0.0004 (5)	0.0057 (5)	−0.0008 (5)
N4	0.0203 (7)	0.0258 (7)	0.0207 (7)	−0.0036 (5)	0.0080 (6)	−0.0055 (6)
N5	0.0441 (10)	0.0371 (10)	0.0324 (9)	0.0111 (8)	−0.0021 (7)	0.0051 (8)
N6	0.0250 (8)	0.0361 (9)	0.0477 (11)	−0.0010 (7)	−0.0008 (7)	0.0112 (8)
N7	0.0320 (10)	0.0777 (15)	0.0622 (13)	−0.0044 (9)	0.0041 (9)	0.0414 (12)
Na	0.0301 (4)	0.0364 (4)	0.0349 (4)	−0.0041 (3)	0.0035 (3)	0.0096 (3)
O1	0.0304 (7)	0.0290 (7)	0.0316 (7)	−0.0025 (5)	0.0100 (5)	0.0066 (5)
O2	0.0317 (7)	0.0307 (7)	0.0285 (7)	−0.0027 (5)	0.0018 (5)	−0.0008 (5)
O6	0.0353 (8)	0.0314 (7)	0.0405 (8)	0.0058 (5)	0.0068 (6)	−0.0025 (6)
O3	0.0248 (7)	0.0324 (7)	0.0421 (8)	−0.0053 (5)	0.0057 (6)	−0.0019 (6)
O4	0.0522 (9)	0.0348 (8)	0.0409 (8)	−0.0053 (6)	0.0209 (7)	0.0043 (6)
O5	0.0538 (10)	0.0521 (10)	0.0553 (10)	−0.0120 (8)	0.0244 (8)	−0.0128 (8)

### Geometric parameters (Å, º)

**Table:**

Cd—N3	2.2076 (13)	C68—H68A	0.9900
Cd—N2	2.2093 (13)	C68—H68B	0.9900
Cd—N4	2.2145 (13)	C69—O4	1.409 (3)
Cd—N1	2.2278 (13)	C69—C70	1.477 (3)
Cd—N5	2.2380 (16)	C69—H69A	0.9900
C11—N1	1.360 (2)	C69—H69B	0.9900
C11—C15	1.411 (2)	C70—O5	1.413 (3)
C11—C12	1.442 (2)	C70—H70A	0.9900
C12—C13	1.356 (2)	C70—H70B	0.9900
C12—H12	0.9500	C71—O5	1.433 (3)
C13—C14	1.445 (2)	C71—C72	1.485 (3)
C13—H13	0.9500	C71—H71A	0.9900
C14—N1	1.362 (2)	C71—H71B	0.9900
C14—C45	1.414 (2)	C72—O6	1.419 (2)
C15—C21	1.402 (2)	C72—H72A	0.9900
C15—C151	1.497 (2)	C72—H72B	0.9900
C21—N2	1.365 (2)	C151—C152	1.379 (2)
C21—C22	1.441 (2)	C151—C156	1.387 (2)
C22—C23	1.347 (2)	C152—C153	1.389 (2)
C22—H22	0.9500	C152—H152	0.9500
C23—C24	1.448 (2)	C153—C154	1.361 (3)
C23—H23	0.9500	C153—H153	0.9500
C24—N2	1.3626 (19)	C154—C155	1.367 (3)
C24—C25	1.398 (2)	C154—H154	0.9500
C25—C31	1.413 (2)	C156—C155	1.385 (3)
C25—C251	1.495 (2)	C156—H156	0.9500
C31—N3	1.3623 (19)	C155—H155	0.9500
C31—C32	1.441 (2)	C251—C252	1.379 (2)
C32—C33	1.356 (2)	C251—C256	1.379 (2)
C32—H32	0.9500	C252—C253	1.378 (3)
C33—C34	1.441 (2)	C252—H252	0.9500
C33—H33	0.9500	C253—C254	1.372 (3)
C34—N3	1.3641 (19)	C253—H253	0.9500
C34—C35	1.418 (2)	C254—C255	1.365 (3)
C35—C41	1.410 (2)	C254—H254	0.9500
C35—C351	1.498 (2)	C255—C256	1.392 (2)
C41—N4	1.365 (2)	C255—H255	0.9500
C41—C42	1.448 (2)	C256—H256	0.9500
C42—C43	1.346 (2)	C351—C352	1.383 (2)
C42—H42	0.9500	C351—C356	1.386 (2)
C43—C44	1.444 (2)	C352—C353	1.389 (2)
C43—H43	0.9500	C352—H352	0.9500
C44—N4	1.365 (2)	C353—C354	1.372 (3)
C44—C45	1.405 (2)	C353—H353	0.9500
C45—C451	1.493 (2)	C354—C355	1.368 (3)
C61—O1	1.412 (2)	C354—H354	0.9500
C61—C62	1.482 (3)	C355—C356	1.389 (3)
C61—H61A	0.9900	C355—H355	0.9500
C61—H61B	0.9900	C356—H356	0.9500
C62—O6	1.423 (2)	C451—C452	1.378 (3)
C62—H62A	0.9900	C451—C456	1.390 (3)
C62—H62B	0.9900	C452—C453	1.408 (3)
C63—O1	1.425 (2)	C452—H452	0.9500
C63—C64	1.492 (3)	C453—C454	1.357 (4)
C63—H63A	0.9900	C453—H453	0.9500
C63—H63B	0.9900	C454—C455	1.358 (4)
C64—O2	1.424 (2)	C454—H454	0.9500
C64—H64A	0.9900	C455—C456	1.375 (3)
C64—H64B	0.9900	C455—H455	0.9500
C65—O2	1.434 (2)	C456—H456	0.9500
C65—C66	1.496 (3)	N5—N6	1.176 (2)
C65—H65A	0.9900	N6—N7	1.163 (2)
C65—H65B	0.9900	N7—Na	2.491 (2)
C66—O3	1.414 (2)	Na—O1	2.5248 (14)
C66—H66A	0.9900	Na—O2^i^	2.5400 (14)
C66—H66B	0.9900	Na—O3	2.5523 (14)
C67—O3	1.425 (2)	Na—O4	2.6076 (15)
C67—C68	1.477 (3)	Na—O6	2.6570 (15)
C67—H67A	0.9900	Na—O5	2.8765 (17)
C67—H67B	0.9900	Na—O2	3.0015 (15)
C68—O4	1.417 (2)	O2—Na^i^	2.5400 (14)
			
N3—Cd—N2	84.58 (5)	O6—C72—H72B	110.2
N3—Cd—N4	83.62 (5)	C71—C72—H72B	110.2
N2—Cd—N4	140.58 (5)	H72A—C72—H72B	108.5
N3—Cd—N1	142.01 (5)	C152—C151—C156	118.53 (16)
N2—Cd—N1	82.88 (5)	C152—C151—C15	120.20 (15)
N4—Cd—N1	83.71 (5)	C156—C151—C15	121.27 (15)
N3—Cd—N5	104.25 (6)	C151—C152—C153	120.61 (18)
N2—Cd—N5	106.81 (6)	C151—C152—H152	119.7
N4—Cd—N5	112.54 (6)	C153—C152—H152	119.7
N1—Cd—N5	113.69 (6)	C154—C153—C152	119.9 (2)
N1—C11—C15	125.56 (14)	C154—C153—H153	120.0
N1—C11—C12	109.12 (14)	C152—C153—H153	120.0
C15—C11—C12	125.33 (14)	C153—C154—C155	120.55 (19)
C13—C12—C11	106.93 (14)	C153—C154—H154	119.7
C13—C12—H12	126.5	C155—C154—H154	119.7
C11—C12—H12	126.5	C155—C156—C151	120.50 (19)
C12—C13—C14	107.16 (14)	C155—C156—H156	119.7
C12—C13—H13	126.4	C151—C156—H156	119.7
C14—C13—H13	126.4	C154—C155—C156	119.85 (19)
N1—C14—C45	125.20 (15)	C154—C155—H155	120.1
N1—C14—C13	108.75 (14)	C156—C155—H155	120.1
C45—C14—C13	126.05 (15)	C252—C251—C256	118.81 (16)
C21—C15—C11	126.58 (14)	C252—C251—C25	120.04 (15)
C21—C15—C151	116.55 (13)	C256—C251—C25	121.13 (15)
C11—C15—C151	116.86 (14)	C253—C252—C251	120.48 (19)
N2—C21—C15	125.71 (14)	C253—C252—H252	119.8
N2—C21—C22	108.92 (14)	C251—C252—H252	119.8
C15—C21—C22	125.35 (14)	C254—C253—C252	120.7 (2)
C23—C22—C21	107.13 (14)	C254—C253—H253	119.7
C23—C22—H22	126.4	C252—C253—H253	119.7
C21—C22—H22	126.4	C255—C254—C253	119.36 (18)
C22—C23—C24	107.51 (14)	C255—C254—H254	120.3
C22—C23—H23	126.2	C253—C254—H254	120.3
C24—C23—H23	126.2	C254—C255—C256	120.36 (18)
N2—C24—C25	125.85 (14)	C254—C255—H255	119.8
N2—C24—C23	108.43 (14)	C256—C255—H255	119.8
C25—C24—C23	125.70 (14)	C251—C256—C255	120.29 (18)
C24—C25—C31	127.32 (14)	C251—C256—H256	119.9
C24—C25—C251	115.96 (13)	C255—C256—H256	119.9
C31—C25—C251	116.71 (14)	C352—C351—C356	117.90 (15)
N3—C31—C25	125.72 (14)	C352—C351—C35	121.20 (15)
N3—C31—C32	108.70 (13)	C356—C351—C35	120.90 (15)
C25—C31—C32	125.33 (14)	C351—C352—C353	120.83 (18)
C33—C32—C31	107.21 (14)	C351—C352—H352	119.6
C33—C32—H32	126.4	C353—C352—H352	119.6
C31—C32—H32	126.4	C354—C353—C352	120.04 (19)
C32—C33—C34	107.18 (14)	C354—C353—H353	120.0
C32—C33—H33	126.4	C352—C353—H353	120.0
C34—C33—H33	126.4	C355—C354—C353	120.28 (18)
N3—C34—C35	125.46 (14)	C355—C354—H354	119.9
N3—C34—C33	108.69 (13)	C353—C354—H354	119.9
C35—C34—C33	125.78 (14)	C354—C355—C356	119.5 (2)
C41—C35—C34	126.39 (14)	C354—C355—H355	120.3
C41—C35—C351	117.37 (13)	C356—C355—H355	120.3
C34—C35—C351	116.22 (14)	C351—C356—C355	121.45 (18)
N4—C41—C35	125.85 (14)	C351—C356—H356	119.3
N4—C41—C42	108.38 (14)	C355—C356—H356	119.3
C35—C41—C42	125.75 (14)	C452—C451—C456	118.98 (18)
C43—C42—C41	107.49 (14)	C452—C451—C45	120.86 (18)
C43—C42—H42	126.3	C456—C451—C45	120.12 (18)
C41—C42—H42	126.3	C451—C452—C453	119.4 (2)
C42—C43—C44	107.31 (14)	C451—C452—H452	120.3
C42—C43—H43	126.3	C453—C452—H452	120.3
C44—C43—H43	126.3	C454—C453—C452	120.3 (2)
N4—C44—C45	125.71 (14)	C454—C453—H453	119.9
N4—C44—C43	108.69 (14)	C452—C453—H453	119.9
C45—C44—C43	125.54 (14)	C453—C454—C455	120.5 (2)
C44—C45—C14	127.44 (15)	C453—C454—H454	119.8
C44—C45—C451	116.95 (14)	C455—C454—H454	119.8
C14—C45—C451	115.61 (14)	C454—C455—C456	120.4 (2)
O1—C61—C62	109.22 (16)	C454—C455—H455	119.8
O1—C61—H61A	109.8	C456—C455—H455	119.8
C62—C61—H61A	109.8	C455—C456—C451	120.5 (2)
O1—C61—H61B	109.8	C455—C456—H456	119.8
C62—C61—H61B	109.8	C451—C456—H456	119.8
H61A—C61—H61B	108.3	C11—N1—C14	108.04 (13)
O6—C62—C61	108.16 (16)	C11—N1—Cd	124.64 (10)
O6—C62—H62A	110.1	C14—N1—Cd	123.68 (11)
C61—C62—H62A	110.1	C24—N2—C21	108.01 (13)
O6—C62—H62B	110.1	C24—N2—Cd	123.66 (10)
C61—C62—H62B	110.1	C21—N2—Cd	125.06 (10)
H62A—C62—H62B	108.4	C31—N3—C34	108.21 (13)
O1—C63—C64	108.58 (15)	C31—N3—Cd	124.20 (10)
O1—C63—H63A	110.0	C34—N3—Cd	126.78 (10)
C64—C63—H63A	110.0	C41—N4—C44	108.13 (13)
O1—C63—H63B	110.0	C41—N4—Cd	125.23 (11)
C64—C63—H63B	110.0	C44—N4—Cd	123.33 (11)
H63A—C63—H63B	108.4	N6—N5—Cd	127.13 (14)
O2—C64—C63	109.03 (15)	N7—N6—N5	177.1 (2)
O2—C64—H64A	109.9	N6—N7—Na	126.76 (14)
C63—C64—H64A	109.9	N7—Na—O1	75.73 (6)
O2—C64—H64B	109.9	N7—Na—O2^i^	160.77 (7)
C63—C64—H64B	109.9	O1—Na—O2^i^	87.25 (5)
H64A—C64—H64B	108.3	N7—Na—O3	79.92 (6)
O2—C65—C66	112.97 (16)	O1—Na—O3	115.42 (5)
O2—C65—H65A	109.0	O2^i^—Na—O3	99.95 (5)
C66—C65—H65A	109.0	N7—Na—O4	85.99 (6)
O2—C65—H65B	109.0	O1—Na—O4	161.06 (5)
C66—C65—H65B	109.0	O2^i^—Na—O4	111.57 (5)
H65A—C65—H65B	107.8	O3—Na—O4	65.03 (5)
O3—C66—C65	109.27 (16)	N7—Na—O6	90.76 (6)
O3—C66—H66A	109.8	O1—Na—O6	63.21 (4)
C65—C66—H66A	109.8	O2^i^—Na—O6	89.42 (5)
O3—C66—H66B	109.8	O3—Na—O6	170.52 (5)
C65—C66—H66B	109.8	O4—Na—O6	112.92 (5)
H66A—C66—H66B	108.3	N7—Na—O5	114.50 (7)
O3—C67—C68	108.84 (17)	O1—Na—O5	123.73 (5)
O3—C67—H67A	109.9	O2^i^—Na—O5	82.30 (5)
C68—C67—H67A	109.9	O3—Na—O5	120.83 (5)
O3—C67—H67B	109.9	O4—Na—O5	59.76 (5)
C68—C67—H67B	109.9	O6—Na—O5	61.56 (5)
H67A—C67—H67B	108.3	N7—Na—O2	92.99 (6)
O4—C68—C67	108.21 (16)	O1—Na—O2	60.99 (4)
O4—C68—H68A	110.1	O2^i^—Na—O2	70.60 (5)
C67—C68—H68A	110.1	O3—Na—O2	61.77 (4)
O4—C68—H68B	110.1	O4—Na—O2	126.07 (5)
C67—C68—H68B	110.1	O6—Na—O2	121.01 (4)
H68A—C68—H68B	108.4	O5—Na—O2	152.50 (5)
O4—C69—C70	108.55 (18)	C61—O1—C63	112.76 (14)
O4—C69—H69A	110.0	C61—O1—Na	120.34 (11)
C70—C69—H69A	110.0	C63—O1—Na	124.29 (10)
O4—C69—H69B	110.0	C64—O2—C65	112.14 (14)
C70—C69—H69B	110.0	C64—O2—Na^i^	117.33 (11)
H69A—C69—H69B	108.4	C65—O2—Na^i^	108.86 (11)
O5—C70—C69	108.5 (2)	C64—O2—Na	101.84 (10)
O5—C70—H70A	110.0	C65—O2—Na	106.55 (10)
C69—C70—H70A	110.0	Na^i^—O2—Na	109.40 (5)
O5—C70—H70B	110.0	C72—O6—C62	112.94 (16)
C69—C70—H70B	110.0	C72—O6—Na	112.55 (13)
H70A—C70—H70B	108.4	C62—O6—Na	105.81 (10)
O5—C71—C72	111.80 (19)	C66—O3—C67	111.37 (15)
O5—C71—H71A	109.3	C66—O3—Na	112.88 (11)
C72—C71—H71A	109.3	C67—O3—Na	109.29 (11)
O5—C71—H71B	109.3	C69—O4—C68	111.87 (16)
C72—C71—H71B	109.3	C69—O4—Na	122.69 (13)
H71A—C71—H71B	107.9	C68—O4—Na	114.65 (11)
O6—C72—C71	107.75 (18)	C70—O5—C71	112.78 (18)
O6—C72—H72A	110.2	C70—O5—Na	110.22 (13)
C71—C72—H72A	110.2	C71—O5—Na	110.93 (12)

Symmetry code: (i) −*x*+1, −*y*+1, −*z*.

### Hydrogen-bond geometry (Å, º)

Cg1, Cg2, Cg3 and Cg11 are the centroids of the N1/C11–C14, N2/C21–C24, N3/C31–C34 and C351–C356 rings, respectively.

**Table:**

*D*—H···*A*	*D*—H	H···*A*	*D*···*A*	*D*—H···*A*
C62—H62*A*···N6	0.99	2.51	3.303 (3)	137
C63—H63*B*···O3^i^	0.99	2.56	3.489 (2)	156
C65—H65*B*···O6^i^	0.99	2.47	3.257 (2)	136
C62—H62*B*···*Cg*1	0.99	2.90	3.555 (2)	124
C63—H63*A*···*Cg*11^ii^	0.99	2.75	3.662 (2)	154
C71—H71*A*···*Cg*3^iii^	0.99	2.88	3.535 (2)	124
C353—H353···*Cg*2^iv^	0.95	2.62	3.459 (2)	147
C454—H454···*Cg*11^iii^	0.95	2.82	3.697 (3)	153

Symmetry codes: (i) −*x*+1, −*y*+1, −*z*; (ii) *x*−1, *y*, *z*; (iii) *x*−3/2, −*y*−1/2, *z*−3/2; (iv) *x*+1, *y*, *z*.

## References

[bb1] Burla, M. C., Caliandro, R., Camalli, M., Carrozzini, B., Cascarano, G. L., De Caro, L., Giacovazzo, C., Polidori, G. & Spagna, R. (2005). *J. Appl. Cryst.* **38**, 381–388.

[bb2] Burnett, M. N. & Johnson, C. K. (1996). *ORTEPII* Report ORNL-6895. Oak Ridge National Laboratory, Tennessee, USA.

[bb3] Byrn, M. P., Curtis, C. J., Goldberg, I., Hsiou, Y., Khan, S. I., Sawin, P. A., Tendick, S. K. & Strouse, C. E. (1991). *J. Am. Chem. Soc.* **113**, 6549–6557.

[bb4] Farrugia, L. J. (2012). *J. Appl. Cryst.* **45**, 849–854.

[bb5] Jentzen, W., Song, X. & Shelnutt, J. A. (1997). *J. Phys. Chem. B*, **101**, 1684–1699.

[bb6] Liu, J.-J., He, X., Shao, M. & Li, M.-X. (2009). *Inorg. Chem. Commun.* **12**, 972–974.

[bb7] Mansour, A., Belkhiria, M. S., Daran, J.-C. & Nasri, H. (2010). *Acta Cryst.* E**66**, m509–m510. 10.1107/S1600536810012080 PMC2979047 21579007

[bb8] Oxford Diffraction (2009). *CrystAlis CCD* and *CrysAlis RED* Oxford Diffraction Ltd, Yarnton, England.

[bb9] Rodesiler, P. F., Griffith, E. A. H., Charles, N. G., Lebioda, L. & Amma, E. L. (1985). *Inorg. Chem.* **24**, 4595-4600.

[bb10] Sheldrick, G. M. (2008). *Acta Cryst.* A**64**, 112–122. 10.1107/S0108767307043930 18156677

